# Feral Cat Globetrotters: genetic traces of historical human‐mediated dispersal

**DOI:** 10.1002/ece3.2261

**Published:** 2016-06-30

**Authors:** Katrin Koch, Dave Algar, Klaus Schwenk

**Affiliations:** ^1^ Biodiversity and Climate Research Centre (BiK‐F) by Senckenberg Naturforschende Gesellschaft and Goethe‐UniversitySenckenberganlage 2560325Frankfurt am MainGermany; ^2^ Science and Conservation DivisionDepartment of Parks and WildlifeP.O. Box 51WannerooWestern Australia6065Australia; ^3^ Molecular EcologyInstitute of Environmental SciencesUniversität Koblenz‐Landau76829Landau in der PfalzGermany

**Keywords:** Australia, commensal, *Felis catus*, Golden Round, Hawaii, invasion, Maritime fur trade, phylogeography

## Abstract

Endemic species on islands are highly susceptible to local extinction, in particular if they are exposed to invasive species. Invasive predators, such as feral cats, have been introduced to islands around the world, causing major losses in local biodiversity. In order to control and manage invasive species successfully, information about source populations and level of gene flow is essential. Here, we investigate the origin of feral cats of Hawaiian and Australian islands to verify their European ancestry and a potential pattern of isolation by distance. We analyzed the genetic structure and diversity of feral cats from eleven islands as well as samples from Malaysia and Europe using mitochondrial DNA (ND5 and ND6 regions) and microsatellite DNA data. Our results suggest an overall European origin of Hawaiian cats with no pattern of isolation by distance between Australian, Malaysian, and Hawaiian populations. Instead, we found low levels of genetic differentiation between samples from Tasman Island, Lana'i, Kaho'olawe, Cocos (Keeling) Island, and Asia. As these populations are separated by up to 10,000 kilometers, we assume an extensive passive dispersal event along global maritime trade routes in the beginning of the 19th century, connecting Australian, Asian, and Hawaiian islands. Thus, islands populations, which are characterized by low levels of current gene flow, represent valuable sources of information on historical, human‐mediated global dispersal patterns of feral cats.

## Introduction

Biodiversity loss through population declines, local and global extinctions of many island endemic species, is largely caused by mammalian invasive species (Atkinson 1985; Fritts and Rodda 1998; Courchamp et al. 2003; Bonnaud et al. 2011; Frank et al. 2014). Feral cats (*Felis catus*) are one of the most widespread introduced invasive predators on islands, having strong negative impacts on the island ecosystems (Fitzgerald 1988; Nogales et al. 2004; Medina et al. 2011; Veitch et al. 2011). Cat invasions led to a major loss in biodiversity of insular birds and mammals as well as local extinction of endemic species (Dickman 1996; Mack et al. 2000; Keitt et al. 2002; Donlan et al. 2003).

The Hawaiian and Australian islands are believed to have been populated by cats most likely through European explorers and later settlers in the early 19th century (Brackenridge 1841; Abbott 2002; Hansen et al. 2007; Hess and Jabobi 2011). After the discovery of the Hawaiian islands by European explorers, for example, Captain James Cook (Cox 1999; King 1984) and following visits by European ships, cats were reported to have spread subsequently through the forests of the Hawaiian islands (Perkins 1903; Rothschild 1893). Since then, cats contributed heavily to the decline and extinction of various endemic Hawaiian bird species (Perkins 1903; Ralph & van Riper III 1985; Smucker et al. 2000; Stone & Scott 1985). From 1840 on, cats occurred across the Hawaiian islands as high‐density stray cat colonies close to human settlements and as isolated feral cat populations in remote montane forests and subalpine areas of Maui and Hawai'i (Brackenridge 1841; Hansen et al. 2007; Hu et al. 2001; Simons 1983; Tomich 1986; Winter 2003).

The European origin of feral cats in Australia has been empirically tested in a previous study, and alternative scenarios, such as possible Asian invasion prior to European settlements, were not supported (Koch et al. 2015). In addition, this study showed that in particular small islands with low or no human populations provided valuable information to reconstruct the history and sequence of the invasion process (Koch et al. 2015). Expanding human migration and trading activities promoted the dispersal of invasive species, introducing them to remote locations around the globe (Elton 1958; Gibson 1992; Greene 1993; Mack et al. 2000; Hess and Jabobi 2011).

Phylogeographic analyses using molecular genetic techniques allow inferences about the origin of a population and its relationship with other populations of the same species (Avise 2009; Bloomquist et al. 2010; MacKay et al. 2013). Routes of introductions can be reconstructed providing information on the pathways of invasion events and the level of connectivity between source and invaded populations (Rollins et al. 2006; Rollins et al. 2009; Schwartz et al. 2007). The ability to identify alien species and to describe their invasion history provides the opportunity to detect and prevent further invasions early on (Rollins et al. 2009). Ongoing intermixing between feral and domestic fancy breed cats, for example, may lead to an increased local genetic diversity and population growth (Dickman 1996; Oliveira et al. 2008; Say et al. 2012). The incorporation of population genetic and phylogeography approaches into various eradication and management campaigns has been found to enhance their success and can assist in recognizing possible positive outcomes of containment efforts (Abdelkrim et al. 2007; Allendorf and Lundquist 2003; Rollins et al. 2006; Schwartz et al. 2007; Veale et al. 2013; Waples & Gaggiotti 2006). This information consequently allows a management design specifically adjusted to population structure and their connectivity to other populations (Estoup & Guillemaud 2010; Rollins et al. 2006; Veale et al. 2013). However, time, frequency, and pathways of cat introductions to islands around the world are mostly unknown.

Since a recent study revealed that islands represent global archives for feral cats’ invasion history (Koch et al. 2015), we analyzed samples from eleven islands from Hawaii, Australia, and Asia using microsatellite and mitochondrial DNA. We addressed the following specific questions regarding the origin, distribution, and variability of feral cat genotypes on these islands: (1) Is the route of introduction represented by historical global trading activities and did they impact the global population structure of feral cats? and (2) Do Hawaiian feral cats originate, as found for Australian mainland and island feral cats, from Europe?.

## Methods

### Sample collection

Feral cat sample collection was carried out on two islands from South‐East Asia (Sulawesi and Malaysia), three Hawaiian islands (Lana'i, Kaho'olawe, and O'ahu), and seven Australian islands (Dirk Hartog Is., Christmas Is., Cocos (Keeling) Is., Tasmania, Flinders Is., Tasman Is., and French Is.). Cocos (Keeling) Island is hereafter referred to as Cocos Island. Trapping, collection of tissue, hair, and blood samples as well as genomic and mitochondrial DNA isolation were conducted as described in Koch et al. (2014). A total of 1800 base pairs (bp) of the mitochondrial ND5 and ND6 region were sequenced using a Bio‐Rad C1000 Thermocycler using conditions as outlined in Koch et al. (2014). For microsatellite analysis, we genotyped at 12 microsatellite loci, which included a gender‐identifying sequence tagged site from the domestic cat Y chromosome SRY gene (Butler et al. 2002; Menotti‐Raymond et al. 2005; Koch et al. 2014). Microsatellite analysis was conducted with all samples except Sulawesi, because samples did not yield sufficient nuclear DNA for adequate genetic analysis.

DNA sequences were determined using an ABI 3730 sequencer and analyzed using Geneious 5.6.6 (Biomatters Ltd L2, 18 Shortland Street Auckland, 1010 New Zealand) software for mtDNA and Genemarker V1.95 (Softgenetics, LLC. 100 Oakwood Ave, Suite 350 State College, PA 16803 USA) software for nuclear fragment analysis.

### Genetic variation and structure

A total of 428 feral cat samples from 11 island populations including 170 previously published Australian/South‐East Asian samples (Koch et al. 2015) and a subset of 41 cats from European locations (Driscoll et al. 2007) were analyzed (Additional file, GenBank: Australian/South‐East Asian dataset: [KP279467–KP279629], European dataset [EF587077.1‐EF587153.1], Table S1A and B). Mitochondrial genetic diversity was based on the number of haplotypes, haplotype diversity (*h*), and nucleotide diversity (*π*) using DNASP V5.1 (Librado and Rozas 2009). We calculated the average number of pairwise differences between population pairs (*G*’’_ST_ values) and their significance estimates with 1000 permutations and 1000 bootstraps (Meirmans and Hedrick 2011) using GENALEX 6.5 (Peakall and Smouse 2012). Oahu (OA) and Malaysian (M) samples were excluded due to small sample size.

An analysis of molecular variance (AMOVA) using mitochondrial data was calculated in ARLEQUIN 3.5 (Excoffier and Lischer 2010). For all analysis performed in ARLEQUIN 3.5 (Excoffier and Lischer 2010), samples were grouped according to four main geographical regions: (1) Australian (OZ); (2) Cocos Island and Christmas Island (CIQ); (3) Hawaii (HI); and (4) Asia (AS).

A Bayesian phylogenetic tree was reconstructed using Beast v1.7.5 (Drummond et al. 2012). Forty‐one European samples from Driscoll et al. (2007) were included in the analysis. The analysis was run 5 × 10^7^ MCMC generations with sampling every 1000th generation. Log files were analyzed using Tracer v1.5, to assess convergence and to confirm combined effective sample size (ESS) >200 for each individual parameter. A maximum credibility tree was subsequently produced using TreeAnnotator v1.7.5. FigTree v1.4.0 was used for graphically display the tree.

A maximum parsimony median‐joining (MP) network was computed using NETWORK version 4.6.1.0 (Bandelt et al. 1999) with frequency >1 criterion being active. Samples from South‐East Asia: Malaysia (M) and Sulawesi (S) were analyzed separately to accommodate eventual differences in haplotype assignment.

Microsatellite data were examined for possible genotyping errors using MicroChecker software (Van Oosterhout et al. 2004). GENEPOP 4.0 software (Rousset 2008) was used to calculate basic population genetic parameters: mean number of alleles per locus (N_A_), expected (H_E_) and observed (H_O_) heterozygosity as well as significance values for deviations from Hardy–Weinberg equilibrium (HWE). Allele frequencies and *F*_IS_ coefficients as a measure of the level of inbreeding were calculated using FSTAT 2.9.3 (Goudet 1995). HP‐Rare (Kalinowski 2005) was used to compensate for differences in sample size and number. Samples South‐East Asia and French Island were excluded due to insufficient data. We tested for evidence of isolation by distance comparing pairwise genetic distances *versus* geographical distances of the islands using the Isolation by distance web service (Jensen et al. 2005). Inference of population structure was based on a discriminant analysis of principal components (DAPC) using the POPPR R‐package (Kamvar et al. 2015).

In order to detect recent population bottlenecks, each population was tested for heterozygosity excess. We used BOTTLENECK version 1.2 software (Piry et al. 1999) and estimated the observed and expected heterozygosity under the two‐phase model with settings of 10% infinite allele model (IAM), 90% stepwise mutation model (SMM) and default settings (30% IAM and 70% SMM) with 1000 iterations.

Fine‐scale population structure was examined by determining the number of private alleles in each population GENALEX 6.5 (Peakall and Smouse 2012). Ancestry structure among populations was studied with STRUCTURE 2.3.4 software (Pritchard et al. 2000). Individuals were assigned to clusters using an unbiased Bayesian approach under an admixture model. Burn‐in and MCMC iteration settings were 50,000 and 100,000, respectively. Each run for *K* was repeated 10 times. STRUCTURE Harvester v 0.6.93 (Earl and vonHoldt 2012) was used to calculate the best number of clusters depending on Δ*K* statistics (Evanno et al. 2005). The software CLUMPP (Jakobsson and Rosenberg 2007) was used to align multiple replicates for the chosen *K* and the DISTRUCT application (Rosenberg 2004) for the graphical display of results.

### Phylogeographic model selection (PMS)

We used MIGRATE‐N 3.4 (Beerli and Palczewski 2010) to choose among competing dispersal hypotheses (Pfenninger and Posada 2002). Three hundred and forty‐one mitochondrial sequences of nine sampling sites were pooled into four geographical groups (Europe, EU; Australian islands, Malaysia/Sulawesi, OZ‐AS; Kaho'olawe, K; Lana'i, L). An additional nuclear dataset of 426 individuals were pooled into five geographical groups (Christmas Island/Cocos Island, CIQ; Kaho'olawe, K; Lana'i, L; Malaysia/Sulawesi, AS; and Australia, OZ. We developed eight phylogeographic hypotheses for the mitochondrial data (Fig. S1) and two migration models for the nuclear data (Fig. S2) based on evidence from historical data and previous studies (Koch et al. 2015). Starting parameters were adapted from Jesse et al. (2011). We ran a burn‐in phase of 10,000 generations and one long chain of 500,000 generations, from which 5000 trees were sampled. The static heating scheme was set to four chains with temperatures 1, 1.50, 3, and 1,000,000. The Bayes factor for all custom‐migration models was calculated and the models with the highest marginal likelihood selected.

## Results

### Genetic population structure and differentiation using microsatellites

We genotyped a total of 428 individuals from 11 sampling locations from Hawaii, Australia, and Asia at 12 polymorphic microsatellite loci; however, one locus (F85) was excluded from population genetic analyses due to the occurrence of null alleles (Van Oosterhout et al. 2004). The expected heterozygosity was moderate to high with a mean of H_E_ = 0.68. Flinders Island (FL) and Tasman Island (TASM) exhibited the lowest genetic diversity with H_E_ = 0.32 and H_E_ = 0.43, respectively (Table 1). The mean number of alleles per population ranged from 1.7 to 14.7 (Table 1). Largest numbers of private allelic richness per population were found for Dirk Hartog Island (DHI) AR = 0.59, and Lana'i (L) AR = 0.53.

**Table 1:** Descriptive statistics for microsatellite data of island cat populations from Australian Hawaii and South‐East Asia (based on 11 loci) including and population sample size (*N*), expected (H_E_) and observed (H_O_) heterozygosity, mean number of alleles (NA), inbreeding coefficient (*F*_IS_), number of private alleles per population (PA) and private allelic richness (AR)

	Populations (abbreviation)	*N*	NA	H_O_	H_E_	*F*_IS_	PA	PA/N	AR
Territorial Islands – Indian Ocean	Christmas Island (CIF)	229	14.7	0.68	0.75	0.09	41	0.17	0.44
	Cocos (Keeling) Island (Q)	50	7.5	0.51	0.64	0.19	4	0.08	0.35
Western Australia – Island	Dirk Hartog Island (DH)	40	9.6	0.73	0.75	0.03	14	0.35	0.59
South eastern Australia	Flinders Island (FL)	3	1.7	0.36	0.32	−0.16			0.21
	French Island (FI)	3	3.7	0.70	0.76	0.10	2	0.6	NA
	Tasmania (TAS)	10	4.8	0.70	0.73		3	0.3	0.49
	Tasman Island (TASM)	5	2.5	0.48	0.43	−0.13			0.12
Hawaii	Kaho'olawe (K)	46	6.7	0.73	0.72	−0.009	3	0.07	0.44
	Lana'i (L)	37	9.7	0.67	0.78	0.14	9	0.24	0.53
	Oahu (OA)	2	3	0.70	0.83	0.22	1	0.5	NA
Asia	Malaysia (M)	3	3.8	0.55	0.78	0.35	4	1.33	NA
	Total	428							

**Figure 1:**
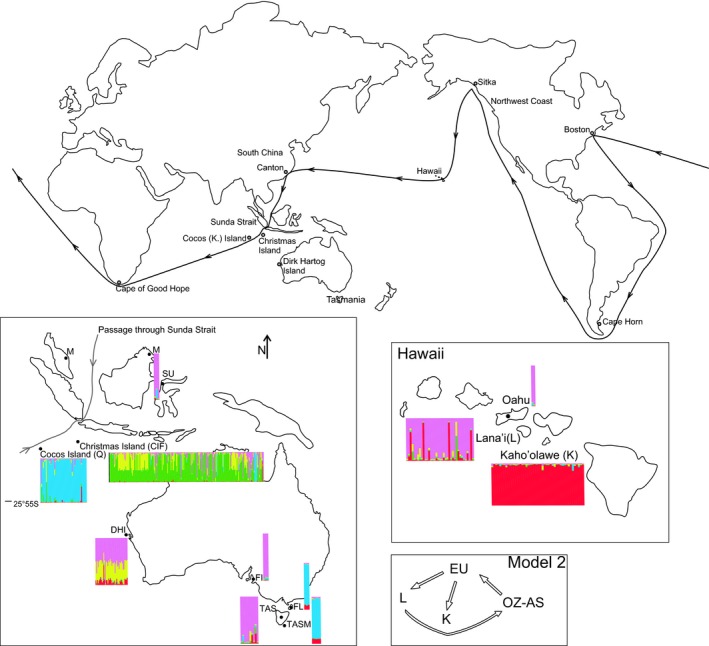
Map of the world representing the main route (Golden Round) used by maritime fur trade (black lines). Boxes show sampling locations in Australia, Hawaii, and South‐East Asia with bars indicating graphical output from STRUCTURE analysis for *K* = 5. Each individual cat is represented by a single vertical line in population's subset plots, which were assigned to their place of origin.

The Bayesian assignment approach revealed a *K* value of five clusters with Kaho'olawe forming a separate single cluster (Fig. 1). This analysis grouped Oahu, Lana'i, French Island, Malaysia, and Tasmania samples together and Tasman Island, Flinders Island, and Cocos Island samples into another cluster. Dirk Hartog Island samples showed strong admixture between clusters.

The analysis of pairwise genetic differences among populations indicated three main clusters: DHI and Kaho'olawe (K), Cocos Island (Q), and Christmas Island (CIF) (PCoA: Fig. S3; 27.5% and 22.9% of variation explained by axis 1 and 3, respectively). Tasmania (TAS), Malaysia (M), and Lanai'i (L) lay with some overlap between clusters. Tasman Island (TASM) and Flinders Island (FL) were distinct from all other populations (Fig. S3). The DACP analysis showed a similar pattern as the PCoA (Fig. S4).

### Mitochondrial phylogeography

**Figure 2:**
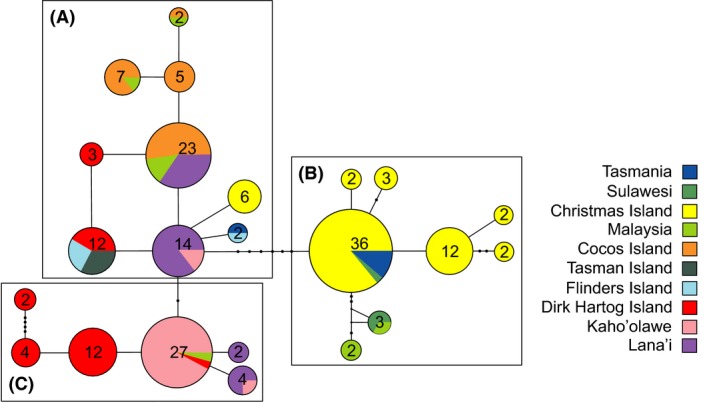
A maximum parsimony median‐joining (MP) haplotype network for Australian, South‐East Asian, and Hawaiian populations consisting of 23 haplotypes divided into three subgroups (A–C). Black dots indicate more than one mutational step. Each additional dot represents one step.

**Figure 3:**
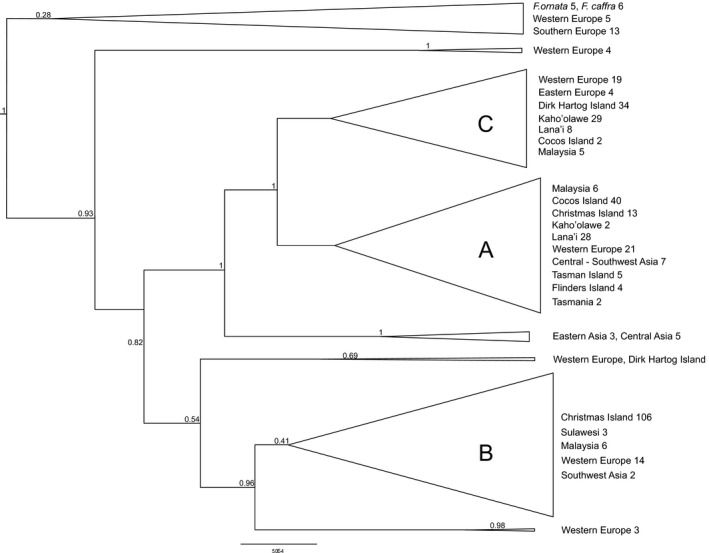
Bayesian phylogenetic tree of mtDNA of cats from Australian, Hawaiian, and South‐East Asian island cat populations. Three clades (A–C) were defined with 95% highest posterior density (HPD) of node ages represented at nodes. The numbers of individuals per location are given. Hyponyms include samples from locations in: England, France, Germany, Scotland: western Europe; Hungary, Serbia: eastern Europe; Spain, Portugal: southern Europe; Kazakhstan, Mongolia, Russia: Central Asia; and Israel, Pakistan, Bahrain, Azerbaijan: southwest Asia.

In total, 2603 base pairs of the mitochondrial genome were analyzed in a total of 300 individuals of nine populations. Altogether a total of 36 haplotypes were detected with numbers ranging from 13 (CIF) to one in populations with small samples sizes (FL, TASM). The mean haplotype diversity was 0.39 with highest values observed for Malaysia/Sulawesi (MS) *h *=* *0.66, TAS *h *=* *0.53, and DHI *h *=* *0.56 (Table 2). Lowest values for haplotype diversity were observed for Cocos Island (Q) and Kaho'olawe (K) with 0.09 and 0.19, respectively. This pattern was also found with mitochondrial nucleotide diversity with MS and TAS showing the highest values (*π *= 0.0026 and 0.0022, respectively). The mtDNA median‐joining haplotype network consisted of 23 haplotypes attributable to three subgroups (Fig. 2). Subgroup A consisted mainly of samples from Cocos Island and Lana'i as well as representatives of all populations except Sulawesi (S), whereas subgroup B consisted of most individuals from Christmas Island together with samples from Malaysia, Sulawesi, and Tasmania. Subgroup C, however, was composed of individuals originating from Dirk Hartog Island, most samples from Kaho'olawe and several individuals from Lana'i and Malaysia. The phylogenetic tree constructed using Bayesian inference (Figs. 3, S5) showed a similar grouping as detected by the haplotype network analysis and the DACP analysis (Fig. S4).

**Table 2:** Measures of genetic diversity of mitochondrial *ND5 + ND6* data from Australian, Hawaiian, and South‐East Asian island cat populations: population sample size (*N*), haplotype diversity (*h*), number of haplotypes (*H*#), and *π* nucleotide diversity

	Populations (abbreviation)	*n*	*H#*	*h*	*π*
Territorial Islands – Indian Ocean	Christmas Island (CIF)	118	13	0.39	0.0015
	Cocos Keeling Island (Q)	43	3	0.09	0.0002
Western Australia – Island	Dirk Hartog Island (DHI)	39	5	0.54	0.0013
South eastern Australia	Flinders Island (FL)	4	1	NA	NA
	Tasman Island (TASM)	5	1	NA	NA
	Tasmania (TAS)	5	2	0.53	0.0022
Hawaii	Kaho'olawe (K)	30	3	0.19	0.0004
	Lana'i (L)	36	3	0.34	0.001
Asia	Malaysia/Sulawesi (MS)	20	5	0.66	0.0026
	Total	300	36		

The comparison of potential routes of introduction using the model selection approach supported a separate introduction of cats from Europe to Lana'i and Kaho'olawe and gene flow from Lana'i to Australia/Malaysia/Sulawesi (Fig. 1, model 2 in Table S2, Figs. S1, S2).

Mitochondrial genetic variation was found to be almost evenly distributed among groups (31.05%), among populations within groups (32.5%), and within populations (36.45%) indicating similar genetic variability between locations, respectively, within populations (Table 3).

**Table 3:** Results of hierarchical AMOVA using mtDNA sequences with abbreviations as in Table 1. Levels of significance are based on 1000 random permutations

Source of variation	df	Sum of squares	Variance components	Percentage of variation	*P* value	Fixation indices
Among groups	6	541.40	1.13626 Va	31.05	0.46515	*F*_CT_ = 0.31054
Among populations within groups	2	14.40	1.18915 Vb	32.50	0.01634	*F*_SC_ = 0.47139
Within populations	292	389.38	1.33352 Vc	36.45	<0.001	*F*_ST_ = 0.63554

Pairwise population comparison showed strong genetic differentiation between samples with *G*’’_ST_ values ranging from 0.15 to 0.89 (Table 4). In particular, Tasman Island (TASM) and samples from Hawaiian islands (K, L) showed high genetic differentiation. Low genetic differentiation is found between TASM and Flinders Island (FL) as well as L and Christmas Island (CIF).

**Table 4:** Genetic differentiation among populations based on microsatellite DNA data (lower matrix pairwise *G*’’_ST_ values; upper matrix *P*‐values) with abbreviations as in Table 1

	CIF	K	L	Q	DH	TAS	TASM	FL
CIF		*	*	*	*	*	*	*
K	0.641		*	*	*	*	*	*
L	0.337	0.481		*	*	*	*	*
Q	0.547	0.731	0.670		*	*	*	*
DH	0.607	0.549	0.521	0.792		*	*	*
TAS	0.431	0.617	0.520	0.663	0.662		*	*
TASM	0.891	0.864	0.860	0.764	0.803	0.833		–
FL	0.684	0.763	0.715	0.527	0.694	0.570	0.156	

Asterisks (*) indicate significant *G*’’_ST_ (>0.05) and (–) indicate nonsignificant differences calculated with 1000 permutations.

**Figure 4:**
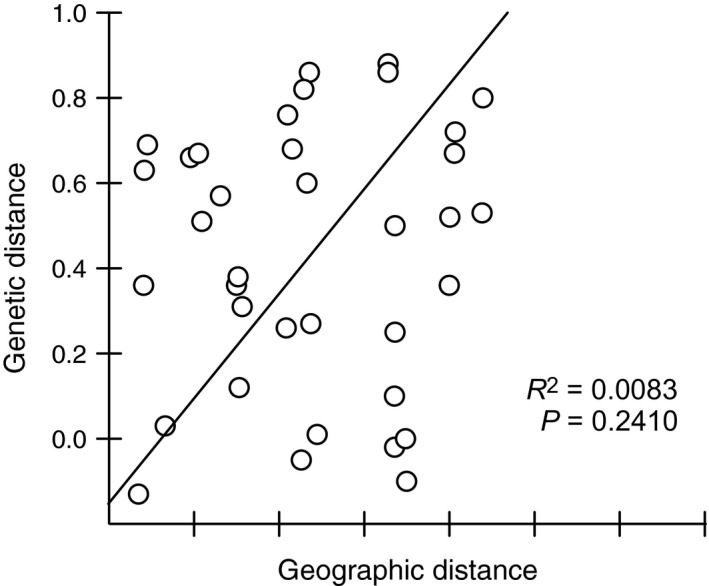
The genetic distance (pairwise *F*_ST_) was plotted against the geographical distance of nine island populations (km). (*R* ^2^ = 0.0083, *P* = 0.2410).

The genetic differentiation (*F*_ST_) among nine populations was plotted against the geographical distance (Fig. 4). The resulting *R* ^2^ showed that only 0.83% of the genetic differentiation (*F*_ST_) was accounted for by geographical distances (*P* = 0.24). No evidence of recent bottlenecks in any of the feral cat populations (*N* > 10) was detected.

## Discussion

During the time of extensive European exploration around 200 years ago, multiple islands including the ones used in this study were invaded by cats (Brackenridge 1841; Abbott 2002; Hansen et al. 2007; Hess and Jabobi 2011). Our analysis of current island populations suggests multiple introductions from different source populations, because we detected no bottleneck effect and an average level of genetic variability in comparison with neighboring mainland populations (Pontier et al. 2005; Hansen et al. 2007; Koch et al. 2015). In particular, Christmas Island (CIF), Dirk Hartog Island (DHI), and Lana'i (L) displayed a high genetic diversity, which was found to be similar to the Australian mainland and European domestic cat populations (A = 8.5, H_O_ = 0.76 and A = 14.2, H_O_ = 0.70, respectively; Pierpaoli et al. 2003; Hansen et al. 2007; Koch et al. 2015).

An overall European origin, especially from central and western European locations, of Hawaiian Island populations is revealed by a balanced grouping of European populations within the three main phylogenetic clusters (A–C in Fig. 3). This pattern is well supported by the model selection approach (Fig. 1, model 2 in Table S2, Figs. S1, S2) providing evidence for gene flow from Europe to Lana'i and Kaho'olawe. Hawaiian populations differentiated into two groups within the phylogeographic tree with the majority of Kaho'olawe and Dirk Hartog Island samples composing a single cluster (subgroup C, Fig. 3). Most of the samples from Lana'i formed a distinct cluster together with samples from Cocos Island, Tasman, and Flinders Island (subgroup A, Fig. 3). High levels of gene flow, which was most likely facilitated by the dispersal of cats through intensive sealing, whaling and pearl trading in Australia at the end of the 19th century, occurred between populations of the third cluster (Tasmania, Christmas Island and Europe, subgroup B, Fig. 3; Koch et al. 2015). Despite genetic differentiation among populations (*G*’’_ST_ = 0.66), some haplotypes were shared among Hawaiian, Australian, and Asian populations and no evidence for isolation by distance of these geographical distant populations was detected (Figs. 2, 4, S4). Individuals carrying these shared haplotypes have populated islands separated by approximately 10,000 km. This population structure and gene flow patterns suggest a strong impact of initial human‐mediated dispersal events on current feral cat populations. The following scenario is consistent with observed phylogeographic patterns: Hawaii was first visited by European Captain James Cook, who died on his second visit 1 year later in 1779 (Beaglehole 1974). Cook's accounts of his voyage encouraged merchants and traders from Britain, Russia, America, and China to visit Hawaii regularly to replenish their supplies and seek replacement crews on their routes between North America and ports of East Asia (Greene 1993). Cocos and Christmas Island inhabitation by Malaysian workers started around 1850 (Green 2007). Malaysian laborers were also appointed in the pearling and whaling industry on Dirk Hartog Island, Flinders Island, and Tasman Island. As some current Australian populations exhibit individual cats that carry South‐East Asian haplotypes, it seems likely that Asian cats were brought to Australian islands during a second wave of invasions (Koch et al. 2015). Subsequently, these Asian cats intermixed with individuals of the founder populations that were of European origin (Koch et al. 2015).

The Maritime fur trade between 1785 and 1841, which traded in sea otters pelts (*Enhydra lutris*) operated on the “Golden Round” trade route around the world (Little 1973; Gibson 1992; Mackie 1997). On this route, most ships would firstly sail from northwest America to Hawaii and then to southern China. On their way back, they would pass Malaysia through the Sunda Strait, Indonesia passing through the Indian Ocean to the Cape of Good Hope, Africa. From there, ships would sail to Boston, northeast America, or Britain and finally travel back to their initial starting point rounding South America at Cape Horn (Little 1973; Gibson 1992; Mackie 1997). Other routes started from India traveling through the Sunda Strait to Hawaii and northwest America (Little 1973; Gibson 1992; Mackie 1997).

We propose that cats originating from South‐East Asia were brought onto the trading ships during landings in Malaysia or stopovers at Cocos or Christmas Island. Both islands were habituated by a European merchant trading various goods; that is, timber and provisions employing Malaysian and South‐East Asian workers (Molloy 1830; Slocum 1901). Thereby it is possible that cats were broad on board of trading ships during stopovers in South‐East Asia. These predictions are consistent with the most likely scenario chosen in the model selection approach (see Fig. 1), indicating that dispersal of cats from Europe to Lana'i and Kaho'olawe and from Lana'i to Australia occurred along a general trade route called the “Golden Round.” Alternative routes, such as bidirectional dispersal along the Golden Round, or source (Europe) sink (all other populations) models, showed lower likelihoods (see Fig. S2). In summary, mitochondrial and nuclear data as well as different analytical approaches (DAPC, model selection and phylogenies) support the expectation that extensive trade in South‐East Asia and Australia as well as regular traffic on routes such as the “Golden Round” shaped the global population structure of feral cats.

Population genetic data of Hawaiian and Australian cats demonstrate that island populations represent a valuable source of information to trace historical European and Asian dispersal routes. Despite the high level of isolation of remote islands (e.g., Hawaii), island populations are surprisingly genetically variable, suggesting rare but multiple invasions from different source populations. The genetic structure and diversity of invasive island populations is dependent on the level of historical and recent gene flow (Allendorf and Lundquist 2003; Frankham 2005; Dlugosch and Parker 2008). Kaho'olawe's cat population separates from other Hawaiian and Australian populations using microsatellite analysis (Fig. 1), but show shared haplotypes with Australian and Hawaiian populations using mtDNA data (Fig. 2). Genetic differentiation among Hawaiian Island populations and genetic isolation of the Kaho'olawe population based on microsatellite markers is assumed to be caused by the low number of human habitats and no public access since its use as a US Army training ground and bombing range in 1941 (Judd 1916; Department of the Navy 1979; Warren and Aschmann 1993). Low human habitation is considered as a limitation for cat introductions and a low number of domestic housecats (Dickman 1996; Oliveira et al. 2008; Say et al. 2012; Koch et al. 2015). Cat populations on the islands did not originate solely from ship landings by traders or explorers, but also presumably as secondary introductions as human commensals from nearby islands. The recruitment and intermixing of domestic and stray animals into a wild population is well documented (Dickman 1996; Oliveira et al. 2008; Say et al. 2012), which leads to population growth and an increased genetic variation (Kolbe et al. 2004; Dlugosch and Parker 2008). This would be also applicable for Lana'i with relatively high genetic diversity and a human population size of approx. 3200 inhabitants with numerous domestic cat owners (US Census 2000, US Department of Commerce). A similar pattern can be observed for Tasmanian cat populations, which were found to group within the Australian mainland cluster possibly representing a recent domestic and stray cat genotype, distributed across the Australian mainland (Koch et al. 2015). We assume that grouping of O'ahu, Lana'i, Tasmania, French Island, Asia, and portions auf Dirk Hartog Island individuals into a shared cluster in the microsatellite analysis are based on the intermixing with domestic fancy breed cats (Fig. 1). These patterns show how the current population structure of feral cats is dependent on recent evolutionary history (e.g., bottleneck, isolation or human‐mediated long‐distance dispersal) and recent intermixing with domestic cats.

In conclusion, we found a mainly European ancestry for cats in Hawaii and low genetic differentiation among cats from Australian islands. This population structure is mainly explained by passive human‐made dispersal during extensive trading in the 19th century such as the “Golden Round.” Drawing inference on the genetic structure and development of invasive species populations, such as the feral cat, is greatly mediated by the degree of human activities (e.g., multiple introductions) with potential gene flow among domestic and feral cats.

## Availability of Supporting Data

The microsatellite data sets supporting the results of this article are available in the Dryad repository [Dryad doi:10.5061/dryad.6t066 (http://dx.doi.org/10.5061/dryad.6t066)]. Sequence data of previously published samples from Australia and South‐East Asia are available in GenBank, [GenBank accessions: KP279467–KP279629, http://www.ncbi.nlm.nih.gov/genbank].

## Conflict of Interest

None declared.

## Supporting information


**Figure S1.** Figures illustrating the phylogeographic model selection as applied to the mitochondrial ND5+ ND6 between Europe (EU), Australia and Southeast Asia (OZ‐AS), Kaho'olawe (K) and Lana'i (L).

**Figure S2.** Figures illustrating the phylogeographic model selection as applied to the nuclear DNA data between Australia (OZ), Tasmanan Island and Flinders Island (TASM‐FL) Tasmania (TAS), Dirk HArtog Island (DHI), Christmas Island (CIF), Cocos (Keeling) Island (Q), Kaho'olawe (K) and Lana'i (L).

**Figure S3.** Principal Coordinates Analysis (PCoA) plot indicating genetic distances between individuals from eleven populations.

**Figure S4.** Inference of population structure of ten island populations based on a Discriminant Analysis of Principal Components (DAPC).

**Figure S5.** Phylogenetic tree of cats based on mtDNA haplotypes obtained in this paper together with those of Driscoll et al. 2007, reconstructed by Bayesian inference with 95% highest posterior density (HPD) represented at nodes.

**Table S1.** A. List of sample locations with abbreviations for sample location and region as well as number of specimens and corresponding geographical coordinates. B. List of European mitochondrial dataset published by Driscoll et al. (2007) with accession numbers and abbreviation for sample region.

**Table S2.** Results of the phylogeographic hypothesis model selection as applied to the mitochondrial *ND5*+ *ND6* data for movements between Europe (EU), Australia/Malayisa/Sulawesi (OZ‐AS), Lana'i (L) and Kaho'olawe (K) (detailed information of phylogeographic models, Additional file, Figure S1–2). The model with the highest mariginal likelihood indicates the model with the best fit to the data (shown in bold).
