# Supplementary material for: Feral Cat Globetrotters: genetic traces of historical human‐mediated dispersal
Source: Ecol Evol. 2016 Jun 30;6(15):5321–32. doi: 10.1002/ece3.2261 (PMC4984506; doi:10.1002/ece3.2261)
Supplement: Supplementary file 1 — Figure S1. Figures illustrating the phylogeographic model selection as applied to the mitochondrial ND5+ ND6 between Europe (EU), Australia and Southeast Asia (OZ‐AS), Kaho'olawe (K) and Lana'i (L). [file ECE3-6-5321-s001.pdf]

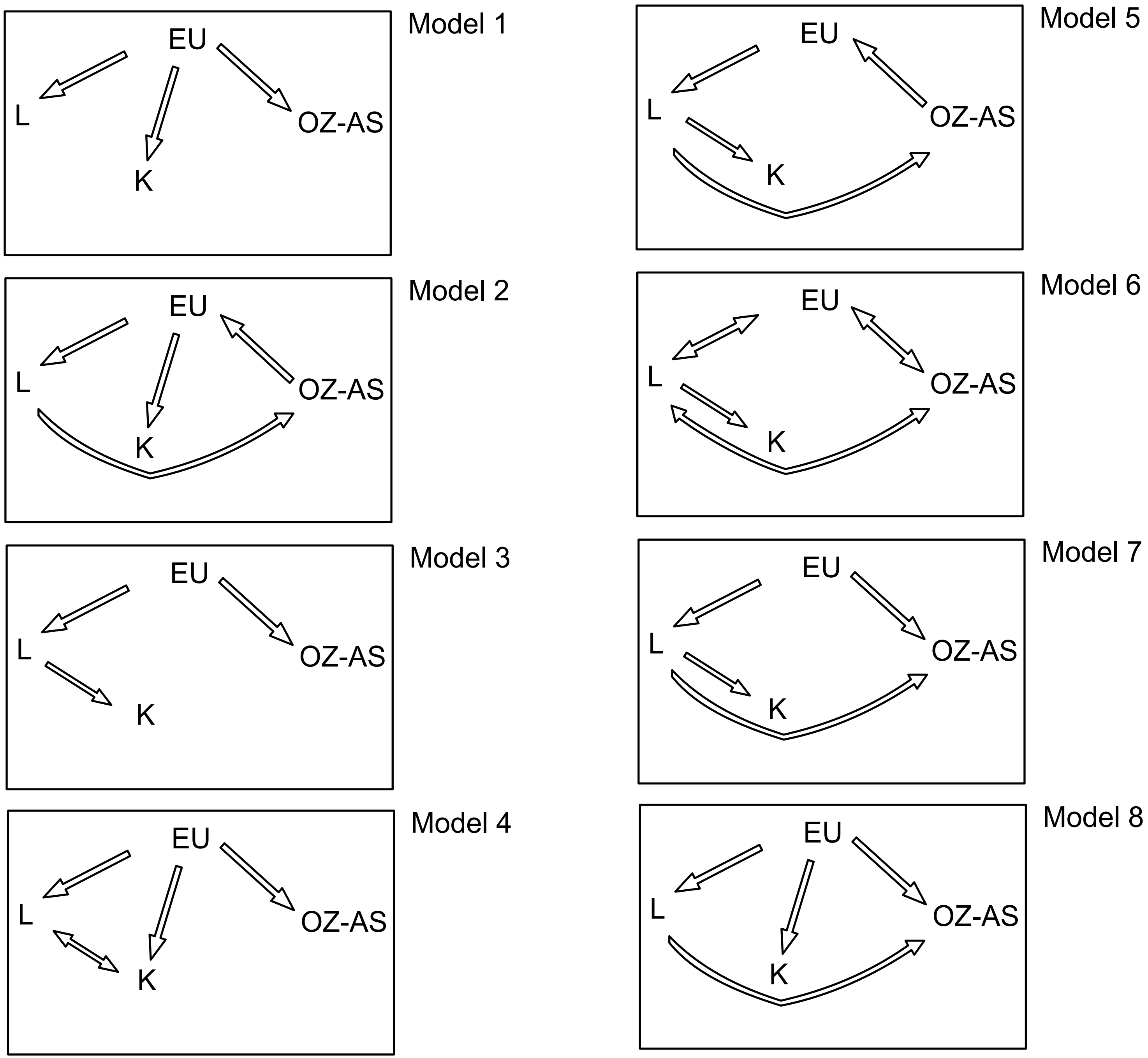

Figure S1. Figures illustrating the phylogeographic model selection as applied to the mitochondrial ND5 + ND6 between Europe (EU), Australia and Southeast Asia (OZ-AS), Kaho'olawe (K) and Lana'i (L).
