# Supplementary material for: Feral Cat Globetrotters: genetic traces of historical human‐mediated dispersal
Source: Ecol Evol. 2016 Jun 30;6(15):5321–32. doi: 10.1002/ece3.2261 (PMC4984506; doi:10.1002/ece3.2261)
Supplement: Supplementary file 2 — Figure S2. Figures illustrating the phylogeographic model selection as applied to the nuclear DNA data between Australia (OZ), Tasmanan Island and Flinders Island (TASM‐FL) Tasmania (TAS), Dirk HArtog Island (DHI), Christmas Island (CIF), Cocos (Keeling) Island (Q), Kaho'olawe (K) and Lana'i (L). [file ECE3-6-5321-s002.pdf]

Model 1

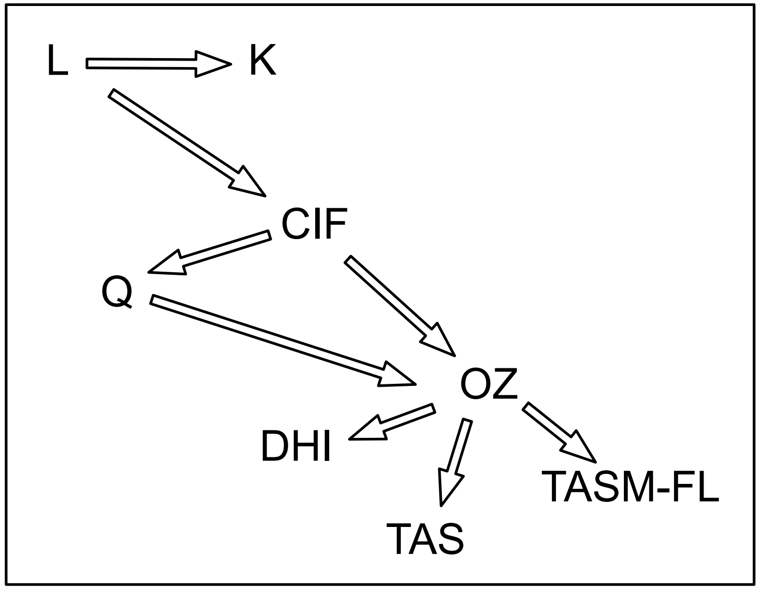

Model 2

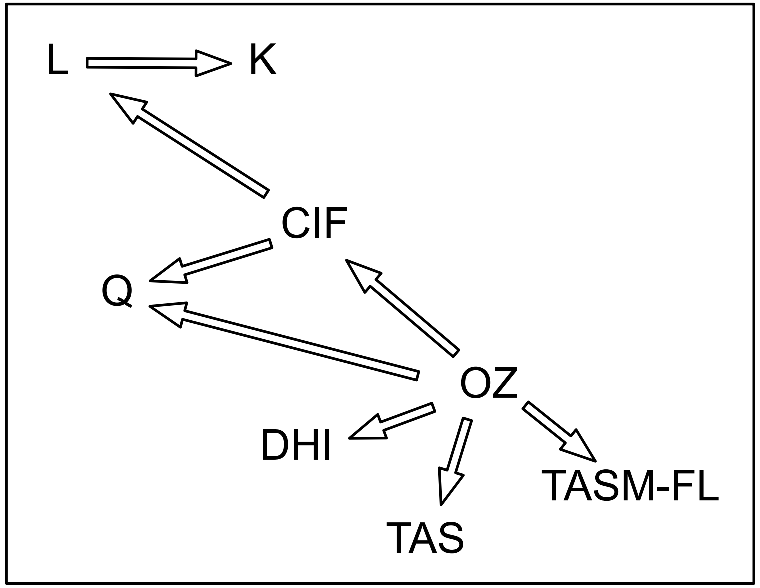

Figure S2. Figures illustrating the phylogeographic model selection as applied to the nuclear DNA data between Australia (OZ), Tasmanan Island and Flinders Island (TASM-FL) Tasmania (TAS), Dirk HArtog Island (DHI), Christmas Island (CIF), Cocos (Keeling) Island (Q), Kaho'olawe (K) and Lana'i (L).
