# Supplementary material for: Feral Cat Globetrotters: genetic traces of historical human‐mediated dispersal
Source: Ecol Evol. 2016 Jun 30;6(15):5321–32. doi: 10.1002/ece3.2261 (PMC4984506; doi:10.1002/ece3.2261)
Supplement: Supplementary file 3 — Figure S3. Principal Coordinates Analysis (PCoA) plot indicating genetic distances between individuals from eleven populations. [file ECE3-6-5321-s003.pdf]

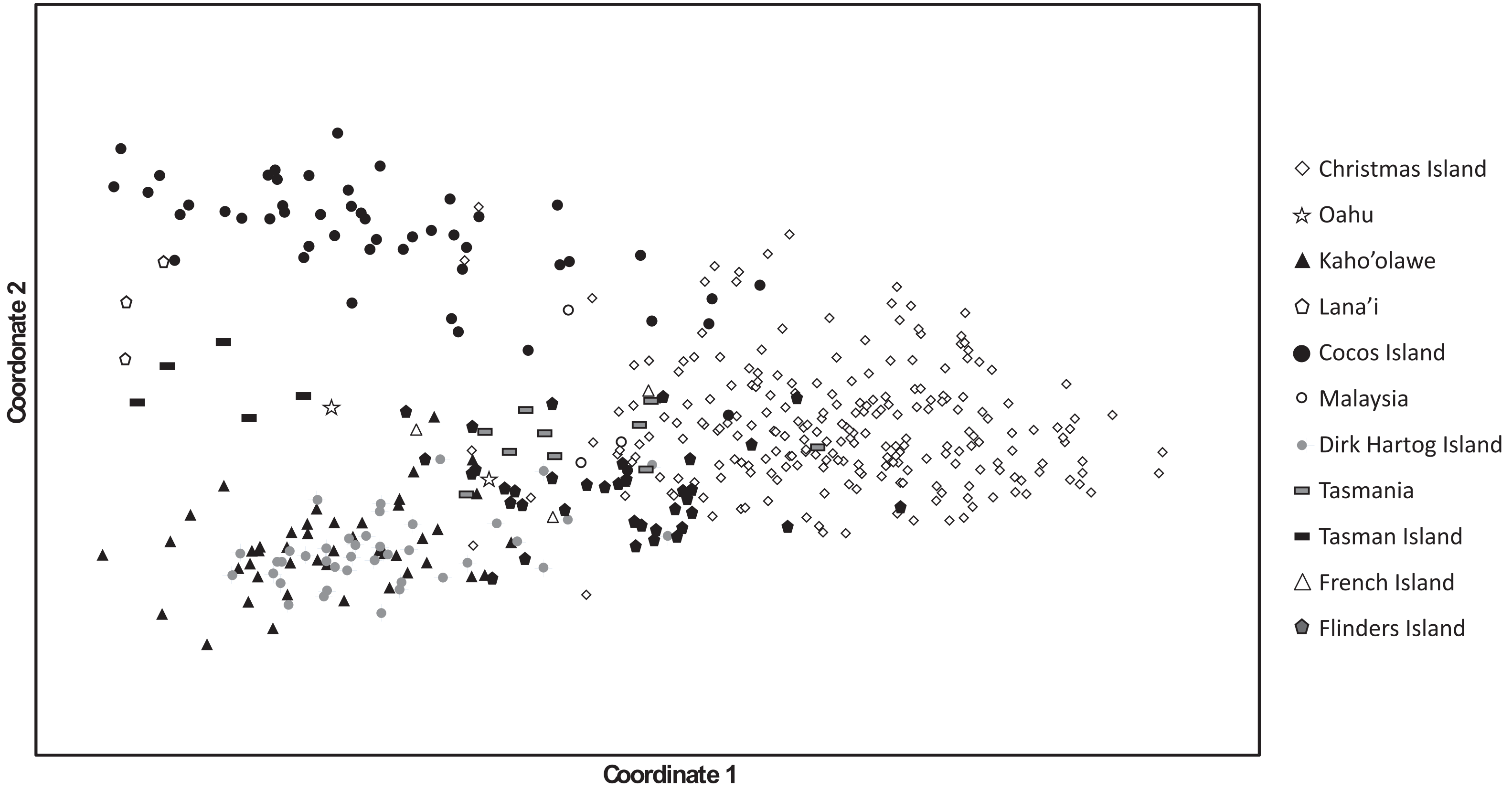

Figure S3. Principal Coordinates Analysis (PCoA) plot indicating genetic distances between individuals from eleven populations. PCoA is based on co-variance distance matrix values using microsatellite data.
