# Supplementary material for: Feral Cat Globetrotters: genetic traces of historical human‐mediated dispersal
Source: Ecol Evol. 2016 Jun 30;6(15):5321–32. doi: 10.1002/ece3.2261 (PMC4984506; doi:10.1002/ece3.2261)
Supplement: Supplementary file 4 — Figure S4. Inference of population structure of ten island populations based on a Discriminant Analysis of Principal Components (DAPC). [file ECE3-6-5321-s004.pdf]

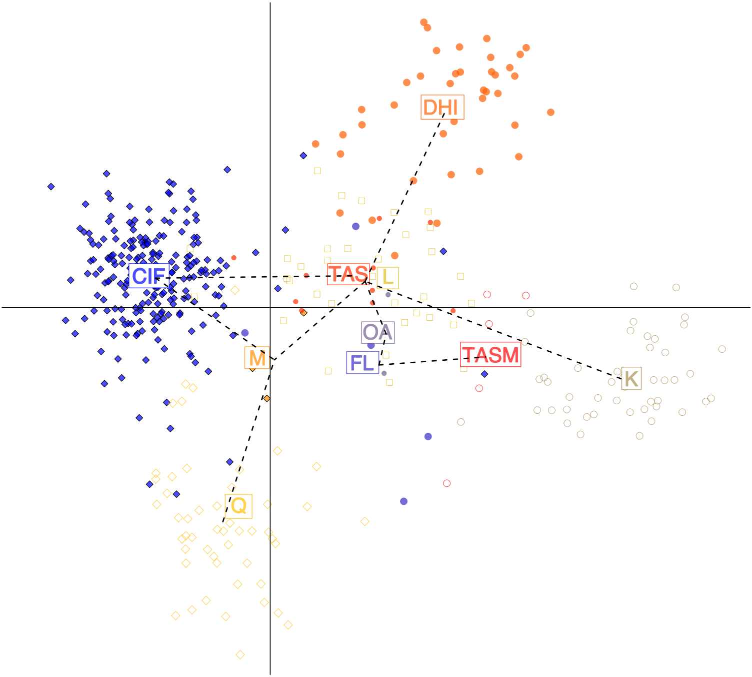

Figure S4: Inference of population structure of ten island populations based on a Discriminant Analysis of Principal Components (DAPC).
