# Supplementary material for: Feral Cat Globetrotters: genetic traces of historical human‐mediated dispersal
Source: Ecol Evol. 2016 Jun 30;6(15):5321–32. doi: 10.1002/ece3.2261 (PMC4984506; doi:10.1002/ece3.2261)
Supplement: Supplementary file 5 — Figure S5. Phylogenetic tree of cats based on mtDNA haplotypes obtained in this paper together with those of Driscoll et al. 2007, reconstructed by Bayesian inference with 95% highest posterior density (HPD) represented at nodes. [file ECE3-6-5321-s005.pdf]

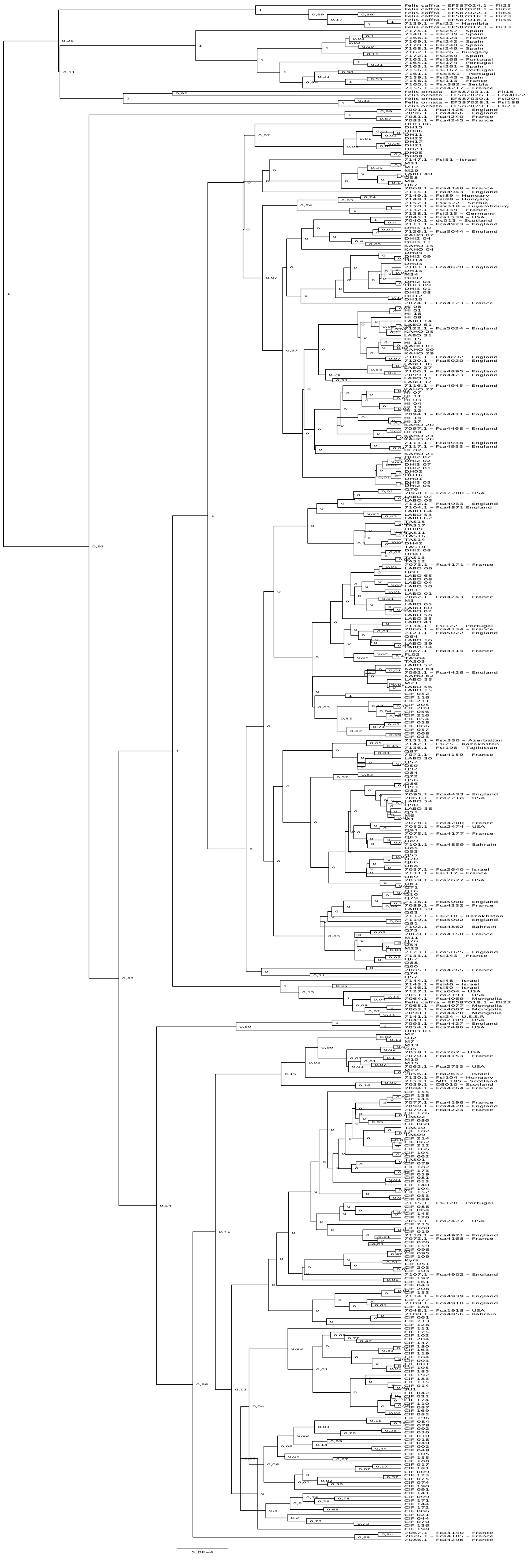

Figure S5. Phylogenetic tree of cats based on mtDNA haplotypes obtained in this paper together with those of Driscoll et al. 2007, reconstructed by Bayesian inference with 95% highest posterior density (HPD) represented at nodes.
