# Supplementary material for: Feral Cat Globetrotters: genetic traces of historical human‐mediated dispersal
Source: Ecol Evol. 2016 Jun 30;6(15):5321–32. doi: 10.1002/ece3.2261 (PMC4984506; doi:10.1002/ece3.2261)
Supplement: Supplementary file 6 — Table S1. A. List of sample locations with abbreviations for sample location and region as well as number of specimens and corresponding geographical coordinates. B. List of European mitochondrial dataset published by Driscoll et al. (2007) with accession numbers and abbreviation for sample region. [file ECE3-6-5321-s006.docx]

**Table S1.** A. List of sample locations with abbreviations for sample location and region as well as number of specimens and corresponding geographical coordinates. B. List of European mitochondrial dataset published by Driscoll et al. (2007) with accession numbers and abbreviation for sample region.

| **A** |  |  |  |  |
| --- | --- | --- | --- | --- |
| Location | Sample location abbreviation | Abbreviation for region | Number of specimens | Latitude/ longitude |
| Christmas Island | CIF | CIQ | 229 | 10.48396 S, 105.635794 E |
| Cocos (Keeling) Island | Q | CIQ | 50 | 12.176745 S, 96.819696 E |
| Malaysia | M | AS | 17 | 5.834056 N, 116.176552 E |
| Sulawesi | SU | AS | 3 | 0.946428 N, 122.338332 E |
| Tasmania | TAS | OZ | 10 | 42.330398 S, 146.066151 E |
| Flinders Island | FL | OZ | 3 | 39.841386 S, 147.927579 E |
| Tasman Island | TASM | OZ | 5 | 43.239045 S, 148.002957 E |
| French Island | FI | OZ | 3 | 38.295343 S, 145.318245 E |
| Dirk Hartog Island | DHI | OZ | 40 | 25.791562 S, 113.038384 E |
| Kaho’olawe | K | HI | 46 | 20.553351 N, 156.608431 W |
| Lana’i | L | HI | 37 | 20.831483 N, 156.887692 W |
| Oahu | OA | HI | 2 | 21.331893 N, 157.789773 W |

| **B** |  |  |  | | |  |
| --- | --- | --- | --- | --- | --- | --- |
| Sample ID | Location | Abbreviation for region | | Accession number |  |  |
| 7077Fra | France | EU | | EF587077.1 |  |  |
| 7081Fra | France | EU | | EF587081.1 |  |  |
| 7084Fra | France | EU | | EF587084.1 |  |  |
| 7158Fra | France | EU | | EF587158.1 |  |  |
| 7131Fra | France | EU | | EF587131.1 |  |  |
| 7132Fra | France | EU | | EF587132.1 |  |  |
| 7155Fra | France | EU | | EF587155.1 |  |  |
| 7086Fra | France | EU | | EF587086.1 |  |  |
| 7133Fra | France | EU | | EF587133.1 |  |  |
| 7166Fra | France | EU | | EF587166.1 |  |  |
| 7087Fra | France | EU | | EF587087.1 |  |  |
| 7138Ger | Germany | EU | | EF587138.1 |  |  |
| 7152Ser | Serbia | EU | | EF587152.1 |  |  |
| 7160Ser | Serbia | EU | | EF587160.1 |  |  |
| 7162Port | Portugal | EU | | EF587162.1 |  |  |
| 7164Port | Portugal | EU | | EF587164.1 |  |  |
| 7130Hun | Hungary | EU | | EF587130.1 |  |  |
| 7149Hun | Hungary | EU | | EF587149.1 |  |  |
| 7167Hun | Hungary | EU | | EF587167.1 |  |  |
| 7140Spai | Spain | EU | | EF587140.1 |  |  |
| 7159Spai | Spain | EU | | EF587159.1 |  |  |
| 7163Spai | Spain | EU | | EF587163.1 |  |  |
| 7168Spai | Spain | EU | | EF587168.1 |  |  |
| 7169Spai | Spain | EU | | EF587169.1 |  |  |
| 7170Spai | Spain | EU | | EF587170.1 |  |  |
| 7172Spai | Spain | EU | | EF587172.1 |  |  |
| 7174Spai | Spain | EU | | EF587174.1 |  |  |
| 7100Bah | Bahrain | EU | | EF587100.1 |  |  |
| 7101Bah | Bahrain | EU | | EF587101.1 |  |  |
| 7102Bah | Bahrain | EU | | EF587102.1 |  |  |
| 7097Eng | England | EU | | EF587097.1 |  |  |
| 7099Eng | England | EU | | EF587099.1 |  |  |
| 7103Eng | England | EU | | EF587103.1 |  |  |
| 7105Eng | England | EU | | EF587105.1 |  |  |
| 7116Eng | England | EU | | EF587116.1 |  |  |
| 7117Eng | England | EU | | EF587117.1 |  |  |
| 7122Eng | England | EU | | EF587122.1 |  |  |
| 7126Eng | England | EU | | EF587126.1 |  |  |
| 7039Scot | England | EU | | EF587039.1 |  |  |
| 7040Scot | England | EU | | EF587040.1 |  |  |
| 7153Scot | England | EU | | EF587153.1 |  |  |
