# Supplementary material for: Feral Cat Globetrotters: genetic traces of historical human‐mediated dispersal
Source: Ecol Evol. 2016 Jun 30;6(15):5321–32. doi: 10.1002/ece3.2261 (PMC4984506; doi:10.1002/ece3.2261)
Supplement: Supplementary file 7 — Table S2. Results of the phylogeographic hypothesis model selection as applied to the mitochondrial ND5+ ND6 data for movements between Europe (EU), Australia/Malayisa/Sulawesi (OZ‐AS), Lana'i (L) and Kaho'olawe (K) (detailed information of phylogeographic models, Additional file, Figure S1–2). The model with the highest mariginal likelihood indicates the model with the best fit to the data (shown in bold). [file ECE3-6-5321-s007.docx]

**Table S2.** Results of the phylogeographic hypothesis model selection as applied to the mitochondrial *ND5* + *ND6* data for movements between Europe (EU), Australia/Malayisa/Sulawesi (OZ-AS), Lana’i (L) and Kaho’olawe (K) (detailed information of phylogeographic models, Additional file, Figure S1-2). The model with the highest mariginal likelihood indicates the model with the best fit to the data (shown in bold).

| **Model** | **Model probability ln(Prob(D/Model)) with Bezier** | **LBF (Bayes factor)** | **Mariginal likelihood (exp)** | **Model-Probability** |
| --- | --- | --- | --- | --- |
| Model 1 | -8570.006 | -417.034 | 7.6592^E-182^ | 7.6574^E-182^ |
| **Model 2** | **-8152.971** | **0** | **1** | **0.999** |
| Model 3 | -8417.279 | -264.307 | 1.6324^E-115^ | 1.632^E-115^ |
| Model 4 | -8438.174 | -285.202 | 1.3743^E-124^ | 1.374^E-124^ |
| Model 5 | -8161.334 | -8.362 | 0.001 | 0.001 |
| Model 6 | -8178.879 | -25.908 | 5.60135^E-12^ | 5.60005^E-12^ |
| Model 7 | -8225.592 | -72.621 | 2.89104^E-32^ | 2.89037^E-32^ |
| Model 8 | -8186.055 | -33.083 | 4.2867^E-15^ | 4.2857^E-15^ |
